# Who learns from whom? Supporting users and developers of a major biodiversity e-infrastructure

**DOI:** 10.3897/zookeys.150.2191

**Published:** 2011-11-28

**Authors:** Irina Brake, Daphne Duin, Isabella Van de Velde, Vincent S. Smith, Simon D. Rycroft

**Affiliations:** 1 Department of Entomology, Natural History Museum, Cromwell Road, London SW7 5BD, United Kingdom; 2 VU-University Amsterdam, Department of Organization Sciences, De Boelelaan 1081, 1081 HV, Amsterdam, The Netherlands; 3 Royal Belgian Institute of Natural Sciences, Vautierstraat 29, 1000 Brussels, Belgium

**Keywords:** Shared knowledge, computer-supported cooperative work, issue tracking, software engineering, e-infrastructures

## Abstract

Support systems play an important role for the communication between users and developers of software. We studied two support systems, an issues tracker and an email service available for Scratchpads, a Web 2.0 social networking tool that enables communities to build, share, manage and publish biodiversity information on the Web. Our aim was to identify co-learning opportunities between users and developers of the Scratchpad system by asking which support system was used by whom and for what type of questions. Our results show that issues tracker and emails cater to different user mentalities as well as different kind of questions and suggest ways to improve the support system as part of the development under the EU funded ViBRANT programme.

## Introduction

Recently, many large research projects have developed e-infrastructures that are used by scientists with varying degrees of IT skills and by developers with sometimes little knowledge of the needs of the users. The key for large user uptake of an e-infrastructure is to address this knowledge gap by encouraging the two groups to talk to each other. Ideally the communication is bidirectional and instructive for both groups. An important question is how to support this type of communication?

With the emergence of the interactive web, Web 2.0, a range of computer supported communication systems have been developed that facilitate learning from and between users and development teams. The present paper investigates to what extent the Scratchpad support services provide learning opportunities for both groups by asking: which support systems are used, by whom and for what type of questions? Additionally, we will reflect on the pros and cons of the different systems. The Scratchpad project has a variety of support services and we will focus on the use of two particular support services, the “help emails” and the “issues tracker”, for which we have access to the usage data.

The results of our study aim to i) increase knowledge on users’ and developers’ needs for information; ii) further improve communication between developers and users; iii) improve the support system performance of Scratchpads and similar initiatives.

In the following paragraphs we give a short background of our research setting, followed by a description of the data and methods used, and conclude with a discussion of the results and formulate recommendations for project management and further research.

## What are Scratchpads?

Scratchpads (http://scratchpads.eu) are a Web 2.0 social networking tool that enables communities to build, share, manage and publish biodiversity information on the Web. Scratchpad sites range in function from supporting the work of societies and conservation efforts to the production and dissemination of species pages and peer reviewed journal articles.

Scratchpads are free and rely on the open source content management system Drupal (http://drupal.org/). The system allows individuals or groups of people to create their own networks supporting their research communities on the Web. The tool is flexible and scalable enough to support hundreds of networks each with their community’s choice of features, visual design, and data. A detailed description of the system architecture and template design of Scratchpads can be found in Smith et al. (2009) and Smith et al. (2011) in this volume. Scratchpads are further developed as part of the EU FP7 funded ViBRANT project (http://vbrant.eu/) and additional support is provided by the NERC funded eMonocot project (http://e-monocot.org/).

As of 7 September 2011, Scratchpads serve 4,299 registered users across 283 sites (see Fig. 1 in Smith et al. in this volume), ranging from academic to citizen-science audiences. These users have generated 374,770 pages of content since Scratchpads were founded in 2007.

## Co-learning and the Web

The very nature of a Web 2.0 environment like Scratchpads makes it possible and imperative that users and developers collaboratively use and build on information systems. Although both still have their own roles and expertise these are highly entangled and benefit from open communication flows. Simply put, users and developers teach and learn from each other about what they need, know and experience when using the system and internalise this knowledge in their day to day work. In this paper we call this co-learning.

The added value of involving users in product design has been widely reported on by Von Hippel et al. (1994, 1995, 2005). They use the concept of “sticky information” to describe value and challenges of integrating local (user) knowledge in product design. Crowston et al. (2008) state that a buffer of active users is a desirable feature in Open Software projects. According to them “active users create a rich support structure and their archived answers form a valuable knowledge base” (p.70). Inspired by Wagner’s (1997) perspective on co-learning, we argue in this paper that this knowledge base could be of use for users as well as for developers. Wagner (1997) formulates co-learning as an agreement between two parties (in his case researchers and practitioners). In a co-learning agreement he states:

Both (parties) are engaged in action and reflection. By working together, each might learn something about the world of the other. Of equal importance, however, each may learn something more about his or her own world and its connections to institutions and schooling (1997, p.16).

With the Web 1.0 e-learning was introduced. Quickly e-courses and e-conferences were made available by institutions that before specialised in offline teaching, very much a one way direction of learning from teacher to student. With Web 2.0 and its integration of interactive technologies, Wagner’s definition can now be applied to offline and online learning settings. Colazzo et al. (2008) describe how the introduction of “virtual-communities” has not only changed the relation between people involved in learning activities but also the technical approach to e-learning. Their argument is that e-learning has evolved into co-learning with “co” referring to “collaborative” and the “community” element of the interactive Web.

Hence support services can have multiple functions for different actors. This may all sound clear-cut but is co-learning an easy process? Perhaps not. For instance Schuler et al. (1993) describe how software development can significantly benefit from genuine communication between developers and users. However, they stress this is not a straightforward process to set up. Potential barriers such as different values, work styles, even languages may hinder the communication (p. 107). Also we know from Bratitsis et al. (2008) that simply making support service available does not ensure that they will be used. In short, co-learning in a Web 2.0 setting appears to have much to offer for innovation and usability of a system but only bears fruit under the right conditions.

In our case study we deal with: a research e-infrastructure; multiple technologies that facilitate learning; and two parties (users and developers) which engage in a co-learning agreement. The learning technologies we refer to are smart services for communication. By analysing the usage data of the Scratchpad support services we aim to measure the presence of co-learning opportunities in the Scratchpad environment. Additionally, we will explore the process of co-learning to better understand mechanisms behind the use. Based on Wagner’s concept of co-learning (1997) we argue that in our case a co-learning opportunity appears every time a message is posted in one of the support services, either by a user or a developer.

For the purpose of the argument that we make in this paper the users and developers are portrayed as distinct communities, while in reality the line between user and developer is often fluid. Some users have a developer’s background and some developers have a research background in the field.

## Support structure

The Scratchpad platform offers a number of support systems. In this paper we will focus on the two support systems most relevant to our research question on co-learning: the request emails (‘contact us’ email and direct mailing to the Scratchpad development team) and issues tracker. The complete Scratchpad support structure is detailed in Appendix 1.

The Scratchpad ‘contact us’ email (scratchpad@nhm.ac.uk) has been active since about August 2008. The emails cover general enquiries about the project, specific help requests, feature requests and bug reports. They are received by the whole Scratchpad development team and are answered by the team member best suited to the task. After an initial contact via the ‘contact us’ email or during training sessions, many requests are sent directly to the personal email address of team members thus making them more difficult to track.

To overcome this lack of overview, the issues tracker (http://dev.scratchpads.eu/project/issues) was implemented in September 2010. This tracker uses a Drupal module and is integrated into the Scratchpad system. Users access the issues tracker via their individual Scratchpad and are automatically logged in with their username. The user can view existing issues or create a new issue for which he/she needs to select whether it is a bug report, feature request or support request. The issue is added to the list and an email is sent to alert the Scratchpad development team. Each time the request is updated an email is sent to the user as well as to the Scratchpad development team. Issues are picked up by the developer responsible for this kind of requests or can be delegated to a certain developer. New issues are marked as “active” and as the issues are dealt with, the status is changed to other values, like “fixed”, “duplicate”, “postponed”, etc.

Users access the issues tracker via a tab on their Scratchpad. This tab also gives the titles of the last ten issues, so that the user can check whether for example a recent bug has been filed already. If this is the case, the user can subscribe to an issues to receive notification about any updates to this issue, thus for example learning how a specific problem can be solved.

As we highlighted above the Scratchpad support systems facilitate a two-way flow of information between users and developers. Although from the outside it might look as if the services cater first of all for the information needs of the users, they help developers in their work as well. Apart from being alerted to bugs, developers use the information gained from requests to improve the usability of the Scratchpad system as well as the support system itself. Additionally, requests for new features influence the decision process for the future development of the system. For example, several users asked for a quick way to simultaneously edit multiple content which led to the development of the matrix editor.

## Data & methods

In the present study, all issues that were raised via the issues tracker (296) in a nine months period between October 2010 and June 2011 were evaluated. This period closely succeeds the start of the issues tracker in September 2010. Additionally, the email help requests sent to the general Scratchpad ‘contact us’ email address (58 requests), or directly to the lead developer (56 requests) and the user support manager (127 requests) were evaluated as well as some of the messages (10 requests) sent directly to other Scratchpad team members (see Appendix 1 for Scratchpad development team roles).

For the issues a matrix was exported from the issues tracker that included the issue ID, date of creation, user name, Scratchpad URL, request category, number of comments, and date of first reply.

Emails were exported from the respective software into a matrix that included the email address of the sender as well as the receiver, date of creation, and the subject and content of the email. In order to be able to compare emails with issues, all emails were sorted into initial request emails (equalling an issue) and replies to these initial requests (equalling issue comments). Each initial request email was given an ID and the Scratchpad URL, request category, number of comments, and date of first reply was was deducted from the text and date of the request email and its replies.

For both systems the number of days until an issue was first replied to was calculated. Additionally, all emails and issues were labeled as posted either by a “user” or a “developer”. For the purpose of this paper all Scratchpad team members, including those involved in support roles, are regarded as “developers” and all Scratchpad users that are not part of the team as “users” regardless of their professional background.

There are three request categories: bug reports are posted if certain features of a Scratchpad don’t work the way they are supposed to work; support requests are posted if a user does not know how to proceed, if he/she would like help in setting up a site, or if he/she would like changes in the deeper structure of his/her own Scratchpad for which he/she does not have the permission; and feature requests are posted if a user would like additional features or functionality added to the Scratchpads as a whole.

## Results

From October 2010 to June 2011, the email service and the issues tracker together facilitated 547 co-learning opportunities between users and developers. Persons who posted issues worked on 43 different Scratchpads, which is 17.3% of all Scratchpads (249 on 30 June 2011). Persons who sent email requests came from 72 different Scratchpads, which is 28.9 % of all Scratchpads. 27 requests were sent from persons without a Scratchpad at the time of emailing.

### Request categories

Both support systems taken together, about half of the requests were support requests, followed by about a quarter bug notifications and one fifth feature requests (Table 1).

**Table 1.** Overview of requests by category and support system. Number of requests posted by users and developers, by request category and by support system (October 2010–June 2011).

**Request category**	**Issues**	**Emails**	**total**
bug	116 (39.2%)	32 (12.7%)	148 (27.1%)
support	77 (26.0%)	211 (84.1%)	288 (52.7%)
feature	103 (34.8%)	8 (3.2%)	111 (20.3%)
total	296	251	547

There is a significant difference in which system was used for which kind or request. The issues tracker was clearly the preferred system for bugs (79.4% of bugs were posted as issues) and features (92.8%), but not for support requests (36.5%). However, there is some overlap between the two systems as sometimes requests moved from one to the other support system: Five emails were follow ups from the issues tracker (all support requests) and 15 issues were posted as a result of email requests (11 support requests, 3 bugs and 1 feature request).

### Pattern of requests over time

**Figure 1.**
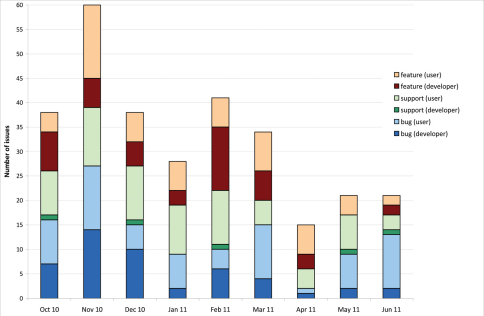
Pattern of issues over time. Number of issues per month divided into request category and each category divided into issues posted by users versus developers (October 2010–June 2011).

In the analysed period 296 issues were raised via the issues tracker (Fig. 1). That is 32.9 per month with a peak in November 2010 due to the follow up for a training course that resulted in many new feature requests, but also in bugs and support requests. In the last three months of the analysed period, the number of issues posted was less, partly because of a drop of issues posted by the developers.

**Figure 2.**
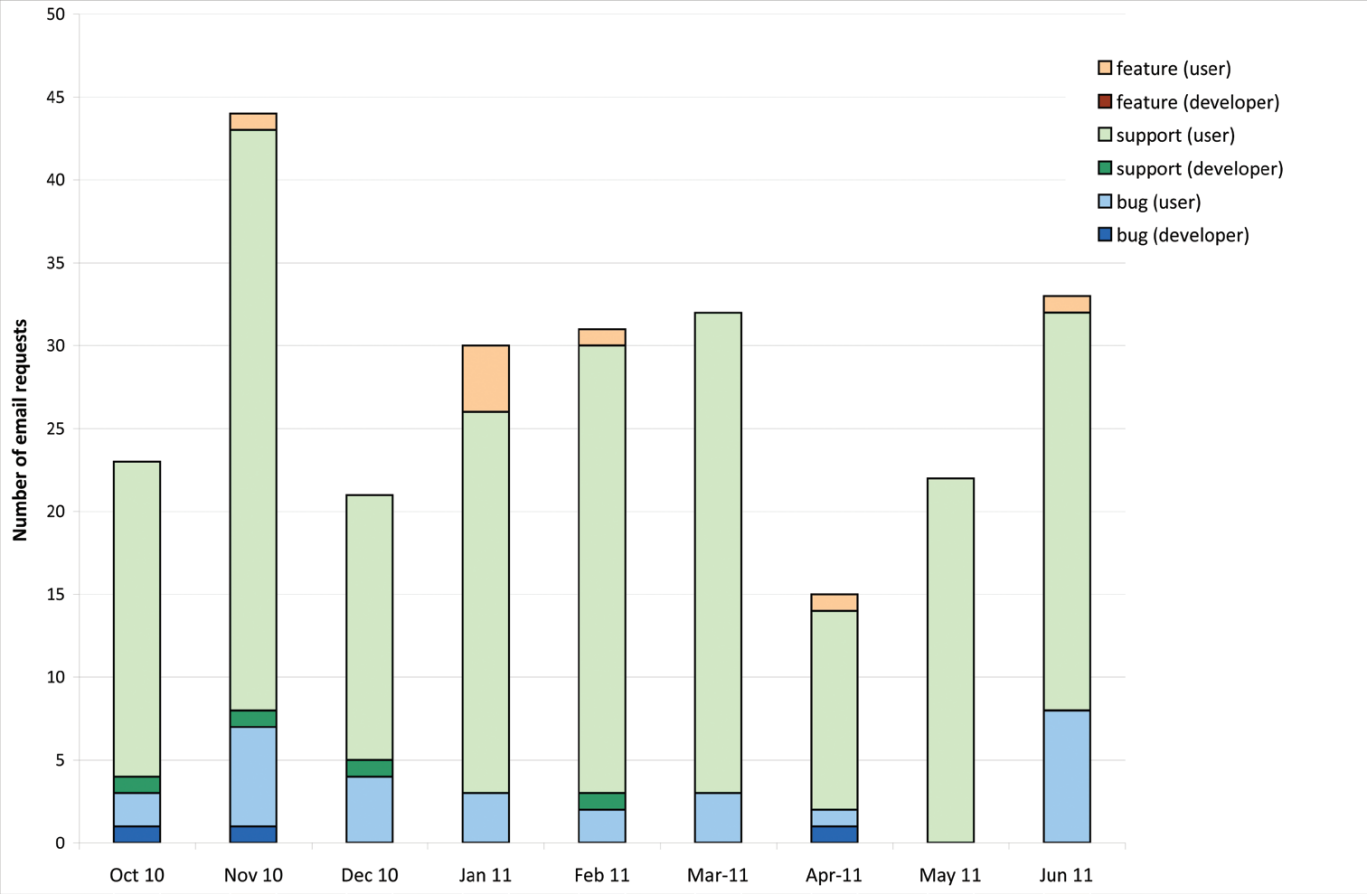
Pattern of email requests over time. Number of email requests per month divided into request category and each category divided into emails sent by users versus developers (October 2010–June 2011).

In the same time period a total of 251 email requests were posted meaning an average of 27.9 requests per month (Fig. 2).

**Figure 3.**
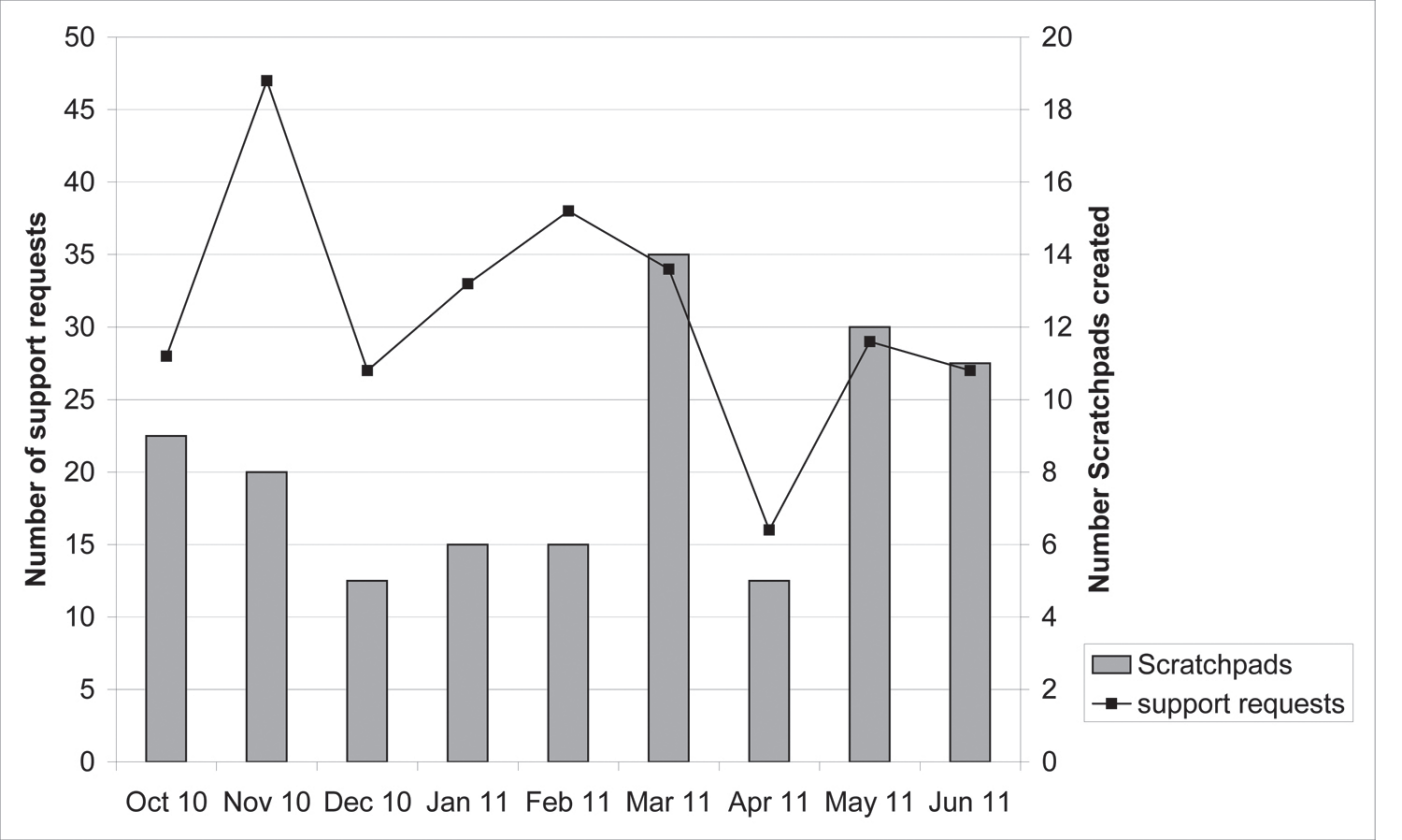
Pattern of support requests and of new Scratchpads over time. Number of support requests posted to both support systems by users and number of new Scratchpads created per month (October 2010–June 2011).

The number of all requests posted to both support systems per month in the analysed period is not related to the number of Scratchpads (see Figure 1 in Smith et al. in this volume) nor to the number of (active) users. However, the number of support requests per month seems to reflect the number of new Scratchpads in the latter part of the studied period (March-June 2011), though not in the earlier part (Fig. 3).

### Pattern of requests by Scratchpad

On average users posted 5.5 requests per Scratchpad (417 requests, 76 Scratchpads). For 22 Scratchpads, five or more requests were posted. Half of these sites were created more than a year before the requests were posted and eight were created shortly before the requests were posted. This pattern is the same if only support requests are considered. So most requests including most support requests are posted by more experienced users.

**Figure 4.**
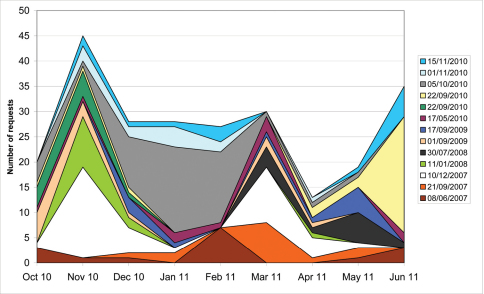
Pattern of requests over time by Scratchpad. Number of requests posted per month (October 2010–June 2011) for Scratchpads for which ten or more requests were posted during the analysed period. In the legend the creation date of the individual Scratchpads is cited.

The pattern of requests over time for individual Scratchpads shows that requests are posted in phases, with periods of high activity alternating with periods of little or no activity (Fig. 4). If requests would have been constant over time, the graph would have depicted straight parallel lines. Instead, large areas in one colour indicate a high request activity on the respective Scratchpad for that time period and small or missing areas indicate low or absent activity.

### User support system preference

When evaluating the 22 users that posted at least five requests, the results show that 15 (68.2%) created more emails than issues, whereas only 7 (17.8) created more issues. Most users used both systems, but 5 (22.7%) clearly prefer using the issues tracker (more than 80% of their requests are posted as issues) and 5 clearly prefer to send emails. Three of the latter did not post a single issue even after having been encouraged to do so.

### Difference between the use of the different support systems by users versus developers

Issues were raised by 49 different persons with an average of 6.0 issues per person. A third (17) of the persons raised only one issue. Six of the persons are developers. However, these six developers posted a significantly higher number of 99 (33.4%) issues (16.5 issues/developer), though it has to be taken into consideration that some of these issues were originally raised by users via email and later on posted by developers to the issues tracker. Out of 296 issues, only 197 were raised by users.

Users and developers also differ in the number of issues posted as different request categories. Support requests are nearly exclusively (93.5%) posted by users, whereas developers posted slightly more bug reports (58.6%) and feature requests (55.3%).

Email requests were sent by 95 different persons with an average of 2.6 emails per person. More than half (57) of the persons sent only one email request. Three of the persons are developers. Only 7 (2.8%) email requests were sent by developers whereas 244 emails were sent by users.

### Request processing amount

On average 3.0 comments were posted per issue and 3.1 per email request (Table 2). Comments are posted by developers as well as users and often represent a discussion thread. In both support systems most comments were posted for support requests (4.0 for issues, 3.3 for emails) and least for feature requests (2.0 for issues as well as emails).

**Table 2.** Number of comments by support category. Number of comments posted by developers and users to the two different support systems by request category (October 2010–June 2011).

**request category**	**number of requests [issues/emails]**	**range of comments [issues/emails]**	**number of comments [issues/emails]**	**average number of comments [issues/emails]**
bug	116/32	0-12/1-9	367/76	3.2/2.4
support	77/211	1-14/0-29	308/686	4.0/3.3
feature	103/8	0-12/1-5	208/16	2.0/2.0
total	296/251	0-14/0/29	883/778	3.0/3.1

### Request processing time

**Figure 5.**
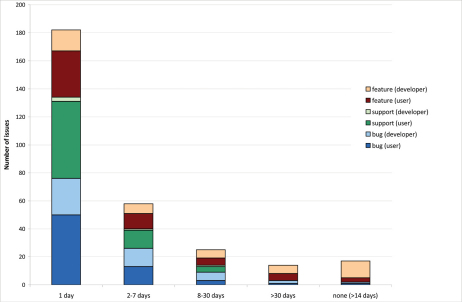
Request processing time for issues. Time lapse between posting of issues and the first reply to this issue divided into request category and each category divided into issues posted by users versus developers (October 2010–June 2011).

182 issues were replied to the same or the following day. 58 issues were replied to within 2-7 days, 25 within 8 to 30 days, 14 after 30 days and 17 have not had any replies by the end of the analysed period (Fig. 5). With “days”, week days are meant, not work days, so within the 2–7 days range issues are included that were replied to the following work day.

Comparing the response rate to issues posted by users versus developers, it becomes obvious that user issues are replied to faster, which is especially true for feature requests and bugs. The major part of the requests that have not been replied to within the analysed period were feature requests posted by developers.

**Figure 6.**
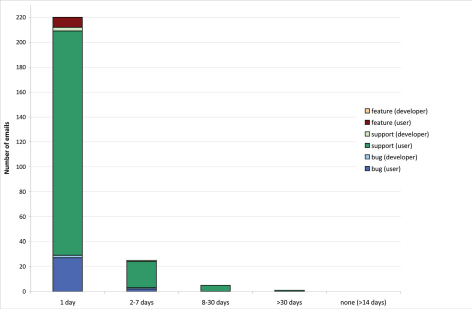
Request processing time for email requests. Time lapse between posting of an email and the first reply to this email divided into request category and each category divided into emails sent by users versus developers (October 2010–June 2011).

220 email requests were replied to the same or the following day (Fig. 6). 25 requests were replied to within 2-7 days, 5 within 8 to 30 days, 1 after 30 days and none have not had any replies by the end of the analysed period.

Comparing issues versus emails, only 61.5% of issues were responded to the same day but 87.6% of emails. Therefore emails are replied to faster than issues.

## Conclusions

In this paper we explored the presence of co-learning opportunities for users and developers in the Scratchpad environment with its various support services and aimed to get a better understanding of the process and mechanisms behind their use. We analysed the usage data of the “request emails” and the “issues tracker”. The results show that the support email service and the issues tracker facilitated 547 co-learning opportunities between users and developers. We think that each request offers a co-learning opportunity because on one hand questions asked by the users are answered by the developers and ways to solve a problem are explained. On the other hand the developers learn about problems with the system and how users work with the system, thus enabling them to improve the Scratchpad system. As a consequence, in early 2012 a new Scratchpad version will be released featuring many enhancements.

**Request categories:** The two support systems are used for different kinds of requests. The issues tracker is the preferred system for bugs and feature requests whereas sending an email is the preferred way for support requests. This reflects the more private nature of support requests, which concern only one Scratchpad, whereas bugs and feature requests usually concern all Scratchpads.

**Pattern of requests over time: **There is a relationship between the number of support requests posted and the number of new Scratchpads per month in the latter part of the analysed period. Assuming that users need more support starting a Scratchpad than later, this would explain the seeming correlation between the number of support requests and number of new Scratchpads in the latter part of the analysed period. The discrepancy in the first part of the analysed period can be explained by the presence of training courses in November (2x), December, January and February, which generated additional support requests due to renewed Scratchpad activity of the training participants. Although we can speculate why these discrepancies happen, further research is needed to better understand what exactly triggers these fluctuations.

**Pattern of requests by Scratchpad:** Requests are posted at various times during the life time of a Scratchpad, not just directly after it was created, even though users often need help in the first months after registering for a new Scratchpad. Usually requests are posted in phases of high activity alternating with low or no activity. Thus it is difficult to predict when periods of higher activity can be expected. The vast majority of requests usually occurred when funding for a person to work on the Scratchpad was available resulting in an extensive use and development of the respective site.

**User support service preference: **The decision on which system to use depends not only on the kind of request (see above), but also on the personality of the user. Most users prefer to write emails, though the number of users that very clearly prefer one system over the other are the same for issues and emails. The reason behind the preference of emails could be that the emails are not published and therefore the barrier to pose what is perceived as “stupid” questions is lower. After the initial contact via the ‘contact us’ email, when a personal contact has been established to a developer it is also for many users more natural to ask questions of this person than to post a request to the more anonymous issues tracker.

**Difference between the use of the different support systems by users versus developers:** An additional factor that influences the way the two support systems are used for different kinds of requests is the role of the person posting the request. Support requests were mostly posted by users, whereas bug reports and feature requests were posted both by users and developers. This reflects the fact that the developers use the issues tracker to keep track of bugs and ideas for new features. Also, often the results of testing of the Scratchpad system as well as problems that arise in other parts of the user support (e.g. training courses, demos) are transferred to the issues tracker. Further research is needed to better understand under what conditions users and developers decide to use the email service and when the issues tracker.

**Request processing amount: **There is a wide range in the number of comments/replies posted in answer to a request. Some requests can be easily fixed and therefore only require one comment notifying of the fix. However, in many cases a request needs to be discussed. Support requests require the most comments/replies because it is often necessary to first get a clear picture of the problem and then to develop customised solutions for which more engagement with the user is needed. This process could be abridged especially for support requests and bug reports by saving the page the user was viewing when entering the issues tracker. This would enable the developer dealing with the request to grasp the problem quicker.

**Request processing time by system:** The time until a request is taken up by one of the developers is different for the two support systems: Emails are replied to much faster than issues. This is partly due to the fact that it takes up to one hour for notifications about new issues to reach the email account of the developers. Emails can also be answered quicker, because it is only necessary to hit the reply button, whereas if a developer receives a notification for an issue, he/she needs to log into a Scratchpad first, go from there to the issues tracker and find and open the correct issue. The process of replying to an issue could be made faster by sending out the notification immediately after a request has been posted and by improving the log in options for developers. Another reason why emails are replied to faster is because most are support requests, which are easier to fix because they usually don’t involve any changes to the Scratchpad system, but just changes to the structure or layout of individual sites.

**Request processing time by role:** An additional factor that influences the time until a request is taken up by one of the developers is the role of the person posting the request. Requests from users are replied to faster than those from developers. This is mostly due to the fact that developers post bug and feature request on the issues tracker for archiving purposes and these requests don’t require immediate attention because they already have been discussed in developer meetings.

## Summary

Based on this study we now have a better view of how two support systems are used by users and developers of Scratchpads and can develop several recommendations for further improvements of the support services itself and the way they are used.

The results underline the importance of offering two support systems, a public system (issues tracker) as well as a private one (emails), to cater for different user mentalities as well as for different categories or requests. A possible advantage of email, privacy of communication, might be important for certain users, but emails are difficult to track for the Scratchpad developers team. Therefore a system should be created whereby emails can be logged into an area of the issues tracker that is private to the Scratchpad team and reply messages should be sent from this area.

Storing the issues tracker and the request emails in one place is also important because they hold a wealth of information on the Scratchpad system. Currently, this information is distributed over several different email archives and the issues system and thereby not accessible to all developers. Having only one archive and tagging all items with keywords would facilitate later tapping of all data. For consistency this tagging should be done by the team.

Although we now have a better view of the presence and process of co-learning opportunities our data did not tell us if actual learning between the two parties has occurred because we did not analyse the content of comments and replies to the requests. Further research using e.g. using survey methods is needed to explore this matter in more depth.

Within the ViBRANT project, networking activities such as workshops, peer-based training courses and cascade training are designed to enhance the use of Scratchpads and to develop a network that will foster long-term sustainability of the user community. Sociological studies of the Scratchpads’ user-base will underpin software development priorities and maximise engagement in the Scratchpads’ community.

## Appendix 1: Scratchpad support structure

### Scratchpad development team

After a conceptual period of several month, the development of the Scratchpad system started with the hiring of a full-time developer in December 2006 and the first Scratchpads were created in January 2007. For the first two years, user support was provided solely by the developer and the project leader, but gradually more experienced maintainers also provided support to colleagues. In January 2010 a user support manager, who is responsible for the help desk, training and the help system, became part of the Scratchpad team. At the time of writing (July 2011) the team consists of the project leader, three developers, and three user support staff (some only part time).

### Support systems

To help with the use of Scratchpads, the first **screencasts **were published in July 2007, followed by **FAQ **in May 2008. Both were available on www.scratchpads.eu. The screencasts were difficult to keep updated and had to be taken down in 2010. In January 2011, the FAQ were replaced by a **help system **that is integrated into the Scratchpads, so that users can find help pages directly on their own site. This help system also contains the manuals for the training courses (see below), providing a more task centered approach than the help pages, which are mostly feature specific.

A mostly empty Scratchpad template, the **sandbox** (http://sandbox.scratchpads.eu/), has been in use since October 2007 to allow Scratchpad maintainers and users to practice using Scratchpads. The sandbox is rebuilt every 6 hours.

The **help desk** was formally started with the appointment of a user support manager in January 2010, but the Scratchpad ‘contact us’ email (scratchpad@nhm.ac.uk) has been active since about August 2008. The help desk deals with all the emails, issues, calls and meetings relating to user support.

As the first feedback system, **UserVoice** (www.uservoice.com) was used together with the EOL Lifedesks (http://www.lifedesks.org) from August 2009 to about April 2010 but was not embraced by the users. This was followed in September 2010 by the current **issues tracker** (see main text).

In order to support and extend the user communities working with Scratchpads, basic and advanced **training courses** are organised. These one-day courses are free of charge and are intended to help current and prospective Scratchpad owners to develop their site building skills, to learn best practices and gain a better understanding of what Scratchpads can do to support research communities.

The training **manuals **are available on the Scratchpads website and have recently been integrated into the help system on each Scratchpad. This allows users to follow the instructions on their own Scratchpad. Additionally, the opportunity is given to do self-training on a **home training site** that can be provided by the Scratchpad development team.

To inform users about new features, bug fixes, training courses, etc. a regular **blog **has been running since January 2010 on the Scratchpads website.

## References

[B1] Bratitsis T Dimitracopoulou A Martínez-Monés A Marcos J Dimitriadis Y (2008) Supporting members of a learning community using Interaction Analysis tools: The example of the Kaleidoscope NoE scientific network. International. Conference ICALT2008, Santader, Spain.

[B2] Colazzo L Molinari A Villa N (2008) From e-learning to “co-learning”: the role of virtual communities. In: Kendall M, Samways B ( Eds) IFIP International Federation for Information Processing 281: 329-338.

[B3] Crowston K Howison J (2006) Knowledge, Technology, & Polio 18 (4): 65-85.

[B4] Schuler D Namioka A (1993) Participatory design: principles and practices. L. Erlbaum Associates Inc. Hillsdale, NJ, USA.

[B5] Smith VS Rycroft SD Harman KT Scott B Roberts D (2009) Scratchpads: a data-publishing framework to build, share and manage information on the diversity of life. BMC Bioinformatics 10 (Suppl 14): S6 10.1186/1471-2105-10-S14-S6 PMC2775152 19900302

[B6] Smith VS Rycroft SD Brake I Scott B Baker E Livermore L Blagoderov V Roberts D (2011) Scratchpads 2.0: a Virtual Research Environment supporting scholarly collaboration, communication and data publication in biodiversity science. In: Smith V Penev L (Eds). e-Infrastructures for data publishing in biodiversity science. ZooKeys 150: 53–70. 10.3897/zookeys.150.2193 PMC3234431 22207806

[B7] Von Hippel E (1994) „Sticky information“ and the locus of problem solving: implications for innovation. Management Science 40 (4): 429-439.

[B8] Von Hippel E Tyre MJ (1995) How learning by doing is done: problem identification in novel process equipment. Research Policy 24(1): 1-12, 10.1016/0048-7333(93)00747-H

[B9] Von Hippel E (2005) Horizontal innovation networks - by and for users. Industrial and Corporate Change 16(2): 293-315, 10.1093/icc/dtm005

[B10] Wagner J (1997) The unavoidable intervention of educational research: a framework for reconsidering researcher-practitioner cooperation. Educational Researcher 26(7): 13-22, 10.3102/0013189X026007013

